# Early Detection of Immune-Mediated Hypophysitis With Use of Checkpoint Inhibitor Immunotherapy

**DOI:** 10.7759/cureus.24291

**Published:** 2022-04-19

**Authors:** Kristena Yossef, Shiva F Naidoo, William Lai, Raghava Reddy Levaka Veera

**Affiliations:** 1 Internal Medicine, Geisinger Health System, Danville, USA; 2 Internal Medicine, Geisinger Health System, Wilkes-Barre, USA; 3 Medical Oncology, Geisinger Health System, Wilkes-Barre, USA

**Keywords:** renal cell carcinoma (rcc), immune mediated side effects, acute hyponatremia, cancer immunotherapy, hypophysitis

## Abstract

Hypophysitis is the inflammation of the pituitary gland with varying effects on hormone function that may be present secondary to the use of certain medications, infections, systemic inflammatory disorders, and other etiologies. Immunotherapy-related hypophysitis is a rare phenomenon. However, it represents an indication of treatment interruption. We report a 60-year-old female with renal clear cell carcinoma on Nivolumab and Ipilimumab (NIVO/IPI) intravenously (IV). After the second cycle of therapy, the patient reported a fall, with associated lightheadedness, dizziness, nausea, vomiting, and hot flashes. The patient's symptoms and history were concerning for hypophysitis, so early treatment and cessation of the checkpoint inhibitors led to the patient's clinical improvement.

## Introduction

Hypophysitis is characterized by inflammatory infiltration of the pituitary gland. It could be primary or secondary to inflammation of nearby structures. The incidence of hypophysitis is 1 new case in 7 million every year, and the incidence of immunotherapy-induced hypophysitis is up to 10-15% of patients receiving anti-cytotoxic T-lymphocyte antigen 4 (anti-CTLA-4) [1]. Neuroradiological studies show enlargement of the pituitary gland, which could be challenging for neuroimaging to differentiate from pituitary tumors [2]. The association with immunotherapy treatment targeting cytotoxic T-lymphocyte antigen 4 (CTLA-4) was described, with the recommendation of discontinuing the treatment in such conditions unless it has been controlled by hormone replacement. Typically, hypophysitis is manifested by clinical symptoms of fatigue and headaches of intensity out of proportion to the size of the lesion and the presence of hypopituitary symptoms. With the increased use of immune checkpoint inhibitors in cancer patients, the occurrence of hypophysitis has become more prevalent [3]. Late detection of hypophysitis, especially that associated with adrenal insufficiency, is associated with high morbidity and mortality [4]. The case presented describes a 60-year-old female with newly diagnosed metastatic renal clear cell carcinoma with lungs and left adrenal gland metastasis. Due to concerns about inferior vena cava involvement, the patient was initially treated with cytoreductive nephrectomy. Subsequently, the patient was started on first-line systemic therapy with IV NIVO/IPI according to Alliance protocol A31704 and phase 3 (PDIGREE study) clinical trial. Early consideration and intervention of the known association of checkpoint inhibitor therapy with hypophysitis helped to prevent later consequences of this disease state.

## Case presentation

A 60-year-old female with metastatic renal clear carcinoma lesions to the lungs and left adrenal gland status post right radical nephrectomy on combined Nivolumab 240 mg and Ipilimumab 85 mg (NIVO/IPI) IV presented to the hospital for a fall at home. Her past medical history was notable for persistent atrial fibrillation on warfarin, chronic systolic heart failure, and essential hypertension.

The patient complained of fatigue and muscle pain after her first cycle of NIVO/IPI. During the pre-procedure evaluation for the second dose, her serum sodium level was 132. Conservative management with rehydration was initiated. After the second cycle was completed, the patient began experiencing nausea, vomiting, and hot flashes. Ondansetron 8 mg IV every six hours as needed was administered, resulting in symptomatic relief.

Eight days later, the patient had a fall at home with progressive weakness and vertiginous symptoms. Repeat serum sodium was found to be 119 mEq/mL, resulting in her admission for acute symptomatic hyponatremia. Her vitals were significant for a blood pressure of 88/59 mmHg and a heart rate of 113 beats per minute with atrial fibrillation with RVR. Metoprolol tartrate 2.5 mg IV was pushed with a subsequent controlled ventricular rate of 73 beats per minute. Vitals were otherwise stable. Endocrinology was consulted and recommended methylprednisolone 80 mg IV push was given at the time.

**Figure 1:**
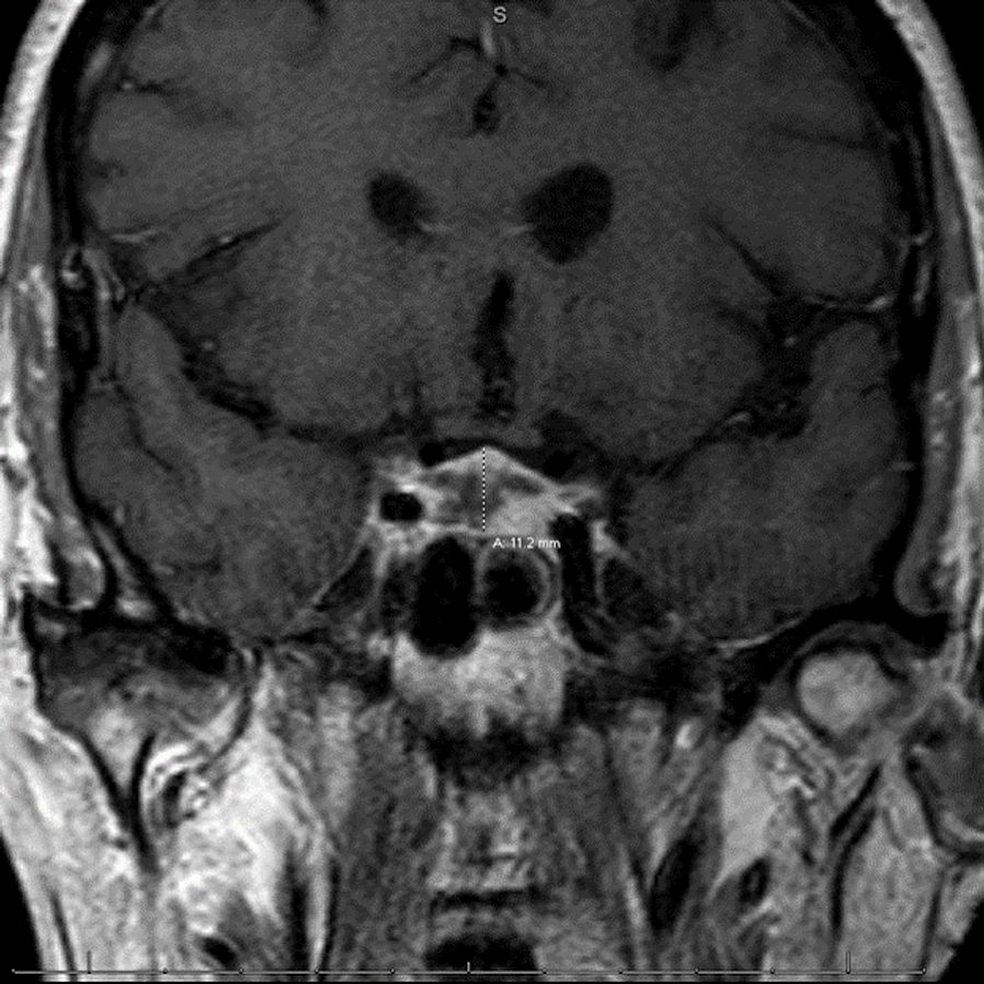
MRI of the brain with contrast T1 weighted coronal cross section highlighting abnormal enhancement and thickening demonstrating inflammation of the pituitary gland and stalk

**Figure 2:**
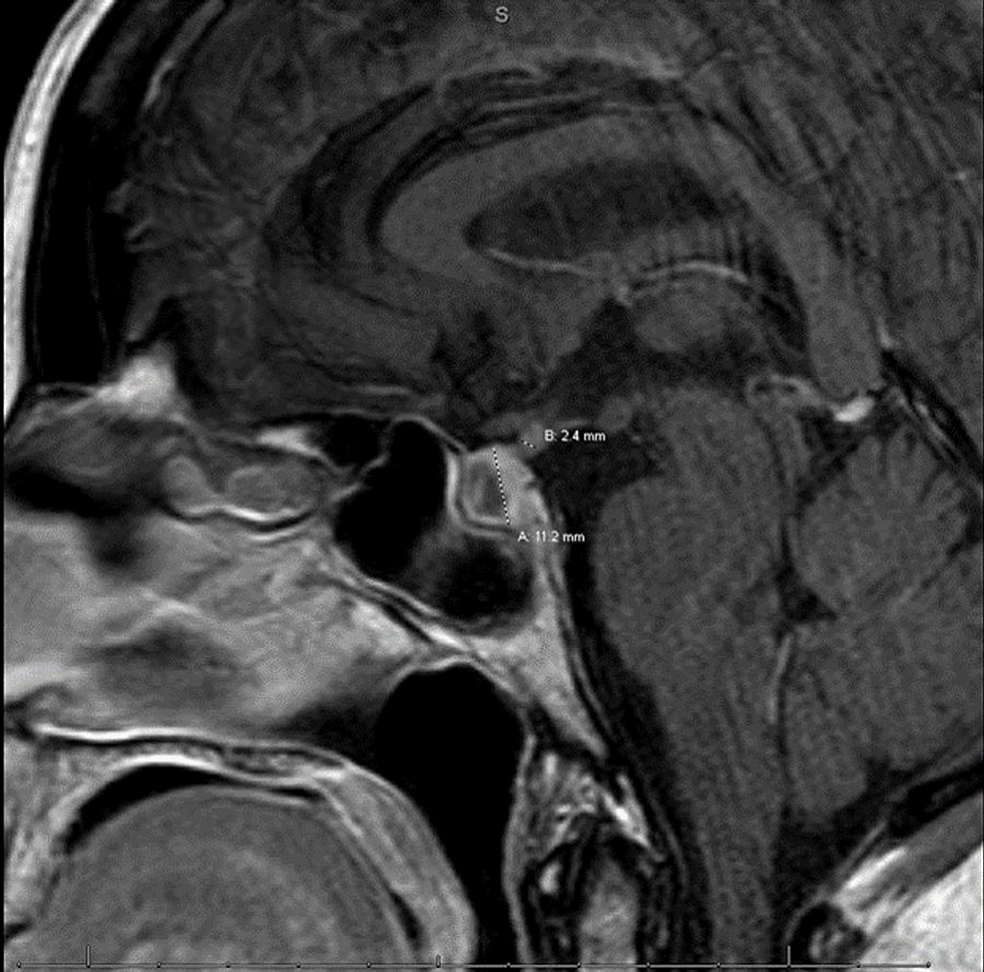
MRI of the brain with contrast T1 weighted sagittal cross section highlighting abnormal enhancement and thickening demonstrating inflammation of the pituitary gland and stalk

Further workup during her hospitalization revealed a morning cortisol level of 1.8 mcg/dL (2.5-19.5 mcg/dL), adrenocorticotropic hormone (ACTH) level of <5.0 pg/mL (0-46 pg/mL), follicle-stimulating hormone (FSH) of 7.4 mlU/mL (25.8-134.8 mIU/mL), luteinizing hormone (LH) of 1.00 mlU/mL (7.7-58.5 mIU/mL), prolactin of 0.1 ng/mL (4.8-23.3 ng/mL), thyroid-stimulating hormone (TSH) of 0.06 ulU/mL (0.27-4.2 uIU/mL), and free T3 of 1.6 pg/mL (2.5-4.3 pg/mL). Urine sodium was found to be <20 mmol/L, and urine osmolality was 386 mOsm/kg. Nephrology was consulted and recommended fluid restriction and tolvaptan 15 mg per os be given. Due to high clinical suspicion for hypophysitis with multiple endocrinological abnormalities and hemodynamic instability, fludrocortisone 50 mcg per os daily, hydrocortisone 15 mg IV in the morning daily, and 10 mg intravenously daily in the evening were given. MRI of the brain with IV contrast was followed by notable findings of abnormal enhancement and thickening of the pituitary stalk, both concerning hypophysitis (Figure 1-2).

Hospital day 3 was significant for clinical improvement, with the resolution of the patient’s dizziness, hot flashes, nausea, and vomiting. On hospital day 4, serum sodium had improved to 132 mEq/mL, and the patient was discharged home with immunotherapy on hold.

## Discussion

Hypophysitis is a rare inflammatory disease estimated to affect 1 in 7 million people [1]. The etiology of hypophysitis is often described as a primary or secondary disease. Primary hypophysitis is classified into three main histopathological categories: lymphocytic, granulomatous, and xanthomatous hypophysitis. Lymphocytic hypophysitis is suggested to be of autoimmune etiology. The etiology of granulomatous hypophysitis and xanthomatous hypophysitis is unknown. Recently, IgG4-related hypophysitis has been regarded as a primary hypophysitis [2]. Secondary hypophysitis often includes immunotherapies, sellar cysts, or pituitary adenomas. Reported immunotherapies with incidences of secondary hypophysitis include the more common CTLA-4 and less often programmed cell death protein 1 (PD-1) therapeutics [5,6]. The etiology of this patient’s disease defines this case as the use of immunotherapies to treat the patient’s underlying cancer increased the clinical suspicion for further workup. The clinical presentation for hypophysitis most commonly includes headaches, vision changes, fatigue, and hypopituitary deficiencies. Immunotherapy-associated hypophysitis occurs in <7% of patients [1]. Diagnosis of hypophysitis is done using laboratory findings to differentiate it from primary adrenal insufficiency and hypothyroidism. Radiography can also be used to help diagnose this acute process, often showing enhancement and swelling of the pituitary gland [7]. In this case, the patient suffered from malaise, hot flashes, hypertension, and tachycardia after receiving two cycles of NIVO/IPI. Subsequent lab work revealed severe hyponatremia. There was a strong concern for adrenal insufficiency, so further adrenal workup to differentiate between primary and secondary adrenal insufficiency was started. The workup included ACTH, random cortisol, prolactin, LH, FSH, TSH, and MRI of the brain with IV contrast. Imaging typically illustrates an enlarged gland with thickened, but not profound stalk deviation on T1 weighted evaluation [8]. The findings, in this case, demonstrated abnormal enhancement and thickening of the pituitary stalk, consistent with hypophysitis (Figure 1-2). The image findings, in combination with the low random cortisol level, low FSH, low LH, low TSH, and low prolactin, further supported the diagnosis of immunotherapy-induced hypophysitis.

NIVO/IPI was the immunomodulator of choice used to treat the underlying renal cell carcinoma. NIVO is a human immunoglobulin G4 monoclonal antibody that binds to the PD-1 receptor and blocks the interaction with programmed death-ligand 1 (PD-L1) and programmed death-ligand 2 (PD-L2), releasing PD-1 pathway-mediated inhibition of immune response, resulting in decreased tumor growth. IPI is a humanized antibody that inhibits T-cell inactivation, promoting the expansion of cytotoxic T cells. Incidences of immunotherapy-mediated hypophysitis post-IPI administration occurred in 1.7% to 1.8% of patients [5].

Acute presentation of hypophysitis, rather than chronic findings, is more likely to warrant intervention [9]. Indications for treatment are mainly centered around reducing compressive effects or hormonal sequelae such as diabetes insipidus subsequent to the diagnosis of hypophysitis [10]. Modalities used in management include surgery, anti-inflammatory therapy, and conservative therapy [11].

Medical management is mainly directed at the correction of pituitary hormone deficiencies, the reduction of pituitary inflammatory changes, and the reduction of mass-related sequelae. Glucocorticoid therapy is the preferred modality currently used, with an initial good response to steroid therapy [11]. In a large cohort, 38% of patients did develop a relapse on steroid therapy [11]. Most commonly, a pulse dose of steroids is preferred. Long-term use of steroids is associated with an increasing risk of adverse effects [11]. In cases with a recurrence of symptoms, immunosuppressive agents or radiotherapy have been used. Commonly used agents include azathioprine. Monoclonal antibodies such as rituximab have been used as well, especially in IgG-4 related hypophysitis [11]. Retrospective studies have previously demonstrated spontaneous resolution without intervention. A single-center trial demonstrated that pulse steroid therapy improved time to pituitary axis recovery and was the preferred modality.

Surgical intervention was previously the most common treatment modality for autoimmune hypophysitis and was performed in 243 (64%) of 379 patients. A transsphenoidal approach was used, and rarely a craniotomy, with the goal of reducing compressive effects on the surrounding structures [12]. The benefits of surgery include a histological diagnosis to further guide management, immediate relief of mass effects, and the further exclusion of tumors. According to the evidence, surgery cannot prevent recurrence [13].

Conservative management with observation can also be considered. A retrospective observational study showed that space-occupying lesions regressed partially in 10 (46%) cases with observation alone, with some lesions remaining unchanged in six (27%) cases and progressing in six (27%) cases. The mean follow-up of 1.2 years demonstrated pituitary function improved in 6 (27%) cases, remained stable in 12 (55%) cases, or worsened in 4 (18%) cases [13]. Consideration of observation may be best decided on the contraindication or complications of other modalities previously discussed, as it may be effective in the interim.

## Conclusions

Hypophysitis is a known but rare autoimmune toxicity of immunotherapy. High clinical suspicion for autoimmune hypophysitis is required to diagnose this condition for prompt management that includes termination of immunotherapy to prevent further deterioration. Early administration of hormone replacement therapy may lead to improved patient-based outcomes.
